# Effect of superficial and deep parasternal blocks on recovery after cardiac surgery: study protocol for a randomized controlled trial

**DOI:** 10.1186/s13063-023-07446-2

**Published:** 2023-07-06

**Authors:** Audrey Jeanneteau, Achille Demarquette, Aymeric Blanchard-Daguet, Olivier Fouquet, Sigismond Lasocki, Jérémie Riou, Emmanuel Rineau, Maxime Léger

**Affiliations:** 1 Département d’Anesthésie Réanimation, Centre Hospitalier Universitaire d’Angers, Angers, France; 2 Service de Chirurgie Cardiaque, Centre Hospitalier Universitaire d’Angers, Angers, France; 3 Département de Biostatistiques Et Méthodologie, Centre Hospitalier Universitaire d’Angers, Angers, France; 4 INSERM UMR 1246, SPHERE, Nantes University, Tours University, France

**Keywords:** Cardiac surgery, Anesthesia, Regional anesthesia, Quality of recovery, Patient related outcomes, Superficial parasternal intercostal plane block, Deep parasternal intercostal plane block

## Abstract

**Background:**

Pain is frequent after cardiac surgery and source of multiple complications that can impair postoperative recovery. Regional anesthesia seems to be an interesting technique to reduce the pain in this context, but its effectiveness in improving recovery has been poorly studied so far. The objective of this study is to compare the effectiveness of two of the most studied chest wall blocks in cardiac surgery, i.e., the superficial and the deep parasternal intercostal plane blocks (SPIP and DPIP respectively), in addition to standard care, versus the standard care without regional anesthesia, on the quality of postoperative recovery (QoR) after cardiac surgery with sternotomy.

**Methods:**

This is a single-center, single-blind, controlled, randomized trial with a 1:1:1 ratio. Patients (*n* = 254) undergoing cardiac surgery with sternotomy will be randomized into three groups: a control group with standard care and no regional anesthesia, a SPIP group with standard care and a SPIP, and a DPIP with standard care and a DPIP. All groups will receive the usual analgesic protocol. The primary endpoint is the value of the QoR evaluated by the QoR-15 at 24 h after the surgery.

**Discussion:**

This study will be the first powered trial to compare the SPIP and the DPIP on global postoperative recovery after cardiac surgery with sternotomy.

**Trial registration:**

ClinicalTrials.gov NCT05345639. Registered on April 26, 2022.

**Supplementary Information:**

The online version contains supplementary material available at 10.1186/s13063-023-07446-2.

## Background

Every year, more than one million people around the world undergo heart surgery by sternotomy [1]. Postoperative pain is frequent after sternotomy, especially in the first 48 h following surgery [1]. The occurrence of this pain is multifactorial [2] and associated with other postoperative complications (e.g., nausea and vomiting, confusion, arrhythmia, myocardial infarction), compromising the early quality of recovery (QoR) and increasing postoperative mortality. In the first postoperative days, this pain is commonly treated with intravenous analgesics including opioids [3], for which side effects may also impair the overall quality of recovery [4].

A multimodal analgesic strategy aiming at sparing opioids, including the use of a peripheral nerve block (PNB) [5], has already shown its usefulness in cardiac surgery [6]. The most recent guidelines of the French Society of Anesthesia and Intensive Care Medicine (SFAR) concerning enhanced recovery after surgery in cardiac surgery highlighted the use of PNB [7]. Among the several techniques of PNB assessed in cardiac surgery, the most promising ones are those based on fascial plane blocks, especially the superficial parasternal intercostal plane block (SPIP) and the deep parasternal intercostal plane block (DPIP), for which frequent names were the pectoral parasternal block [8, 9] and the transversus thoracic muscular plane block [10–12] respectively before the recent standardized nomenclature proposed by the ASRA-ESRA Delphi consensus [13].

In comparison with the SPIP, the DPIP allows the injection of local anesthetic closest to the anterior branches of the intercostal nerves and may be theoretically more efficient. However, there is currently one randomized trial only comparing SPIP and DPIP, which found no difference between the two blocks [14]. Furthermore, while all of these evaluations have focused mainly on pain intensity and opioid consumption, there are no study assessing the impact of the use of a SPIP or a DPIP on the early postoperative quality of recovery.

We aim to compare the effectiveness of the use of a SPIP or a DPIP in addition to standard care, in comparison with standard care without regional anesthesia on the early postoperative QoR after cardiac surgery with sternotomy.

## Methods

**Fig. 1:**
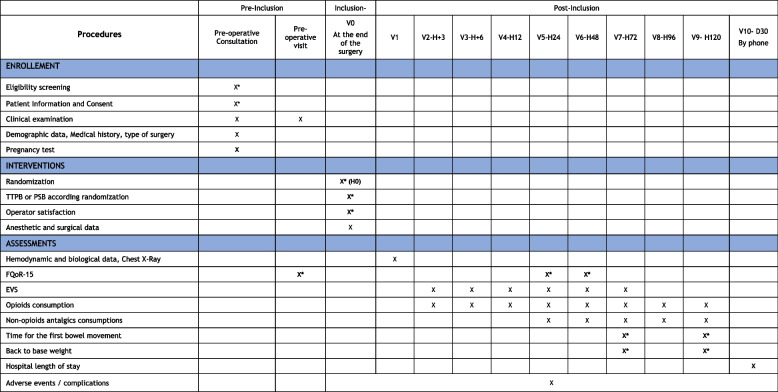
Standard Protocol Items: Recommendations for Interventional Trials (adapted from SPIRIT figure). Abbreviations: NRS, numerical rating scale, FQoR-15, French version of the QoR-15; V1/2/../9, visit 1/2/../9; H + 3/…/ + 120, 3/…/120 h at surgery; D30, 30 days at surgery

**Fig. 2:**
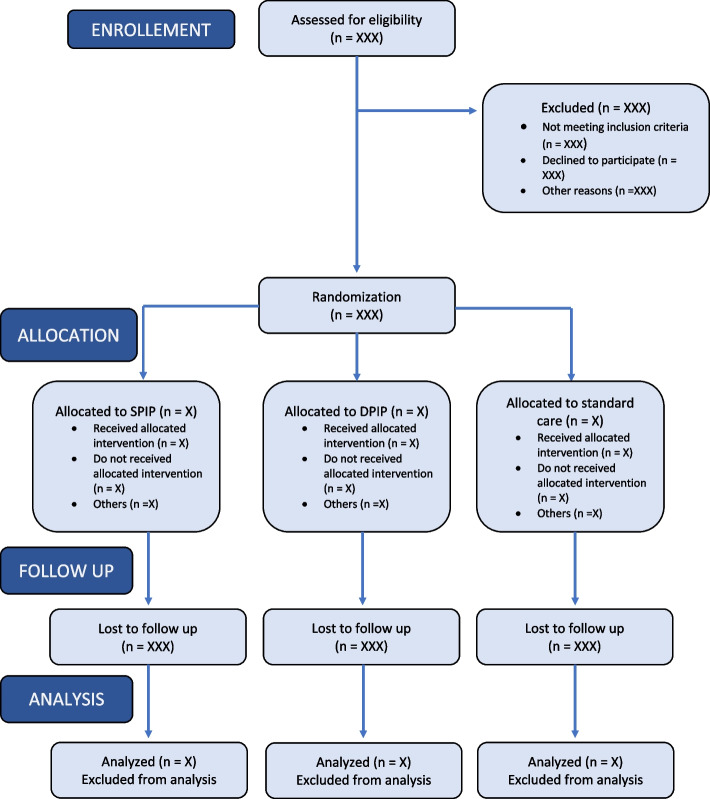
Flow chart

This is a superiority, single-center, single-blinded, controlled, randomized trial in which patients undergoing cardiac surgery by sternotomy will be included. The patients will be randomized into three groups, either in the SPIP group (receiving a SPIP in addition to standard care), the DPIP group (receiving a DPIP in addition to standard care), or the standard group (receiving standard care but no regional anesthesia). The study will be conducted at the Angers University Hospital (Angers, in France). This trial is designed according to the elements of the standard protocol (SPIRIT guidelines). Figures 1 and 2 provide an overview of the study plan.

The French Institutional Review Board North-West IV (Lille, France, reference number: 2022-A00238-35) and the National Agency for Drug and Health Product Safety have approved the study protocol (2022-A00238-35) in its version 4. Patients will provide written consent for participation. The study will be conducted in accordance with the Declaration of Helsinki.

### Participants

Eligible participants are adults (≥ 18 years old) who are going to have cardiac surgery with sternotomy. Eligible patients must be French-speaking and must have the sufficient cognitive ability to complete a questionnaire. Exclusion criteria are known hypersensitivity to local anesthetics, redo heart surgery, emergency surgeries, septic context (endocarditis, intravascular device infection), weight less than 30 kg, psychiatric disorder or severe cognitive impairment hampering assessment by questionnaires, pregnant/breastfeeding/parturient women, persons deprived of their liberty by judicial decision or subjected to psychiatric treatment under duress, and inclusion in another interventional study modifying postoperative pain management. Inclusion will be confirmed at the end of the surgery (i.e., time of the skin suture), in the absence of hemodynamic instability (defined here as a norepinephrine infusion equal or greater than 1 μg/kg/min) and in the absence of bleeding requiring immediate surgical revision.

### Information of patients

During the anesthesia consultation (about 2 weeks before surgery), investigators will invite the patients to participate after verification of the inclusion/exclusion criteria. Patients will receive complete information in faithful terms and understandable language concerning the objectives, the required follow-up, the risks, the safety measures, and the rights to refuse to participate or stop the study at any time. A written informed consent will be signed by both the investigator and the patient. If a longer reflection time is desired, the proposal to participate in the study will be again done to the patient during the pre-surgical visit (the day before or the day of surgery).

### Randomization and blinding

The anesthesiologist in charge will perform the randomization at the end of the surgy in the operating room. The included patients will be randomized using a 1:1:1 ratio in one of the three groups (SPIP group, DPIP group, or standard group). The randomization will be stratified on the achievement or not of an internal thoracic artery graft, as patients having cardiac surgery with internal thoracic arteries are more susceptible to present postoperative acute or chronic pain [15]. The investigators will use the Ennov Clinical® software and will not know the stratification process to avoid guessing the next allocation.

The anesthesia team in charge of the patient in the operating room will be unblinded to the allocation group. Conversely, patients will not know in which group they have been allocated since the block (SPIP or DPIP) will be performed at the end of the surgery, under general anesthesia. To maintain the blinding for the medical and paramedical team in charge of the patient in the intensive care unit, a skin dressing in accordance with the puncture zone will be used for all patients, even for those in the standard group.

### Intervention

This study aims to compare the added effect of parasternal intercostal plane block (SPIP or DPIP) to postoperative standard care. SPIP and DPIP will be performed under general anesthesia in the operating room at the end of the surgery, once the skin closure has been finalized. For skin disinfection, we will use the same antiseptic as for the surgical procedure. The anesthetist performing the procedure will wear sterile gloves, with a mask and a surgical cap. According to the French Society of Anesthesia and Intensive care Medicine (SFAR) recommendations [16, 17], an ultrasound protection and sterile ultrasound gel will be used to perform the block under ultrasound guidance (sagittal section, with a 7.5 MHz ultrasound probe). A 50-mm regional needle with a diameter of 22G will be used. For both types of blocks, the anesthetist should position the probe next to the sternum and laterally move the probe to identify the internal thoracic artery using the color Doppler. When this artery is observed, both techniques consist in puncturing in the craniocaudal plane at 2 cm from the sternum laterally, next to the space between the 4th and 5th ribs.

For the DPIP, the injection is performed between the internal intercostal muscle and the transverse thoracic muscle (20 mL of 0.2% ropivacaine). The lowering of the pleura during the injection is a quality sign [18]. The same injection has to be performed on the other side of the sternum. For the SPIP, the same process is realized but the injection of ropivacaine should be performed between the major pectoral muscle and the external intercostal muscle (20 mL of 0.2% ropivacaine on each side).

Each patient in each of the three groups will be operated on under general anesthesia and will receive standard management with a radial artery catheter for continuous blood pressure monitoring, two peripheral venous catheters, a central venous catheter (right or left internal jugular catheter), and a temperature-sensing indwelling urinary catheter. The anesthetic induction will be performed using total intravenous anesthesia (TIVA) as follows: propofol with plasma target at 2–4 μg/mL, sufentanil with brain target at 0.4–1 ng/mL, an intravenous (IV) bolus of atracurium 0.3 to 0.6 mg/kg, and dexamethasone 8 mg IV. For the anesthesia maintenance, the sufentanil brain target will be set at 0.2–0.8 ng/ml, depending of the different times of the surgery, and inhaled sevoflurane will be used with a minimum alveolar concentration (MAC) objective of 1 during the non-cardiopulmonary bypass period although propofol TIVA only will be used during the cardiopulmonary bypass period.

At the end of the surgery, the patient will be transferred to the intensive care unit (ICU) under intravenous propofol sedation, as usual. The pain management protocol in ICU is detailed in the supplementary material (Table S.1).

### Outcomes

#### Primary outcome

The primary endpoint is the early postoperative QoR, assessed via the French version of the QoR-15 questionnaire score (FQoR-15) [19] and measured at 24 h after the surgery (H24). The Quality of Recovery-15 (QoR-15) is one of the most reliable and reproducible tools for assessing the QoR after surgery [20–22]. The use of this tool as an endpoint has been recommended by a recent international consensus [23] and has already been measured in previous randomized trials [24].

The FQoR-15 is obtained via a self-administered questionnaire, consisting of 15 items scored on an 11-point scale with an overall score (sum of each item) ranging from 0 to 150 (0 for very bad recovery, 150 for excellent QoR). The questionnaire will be given by the nurses to the patient in ICU on a paper sheet that will be filled out by the patient.

#### Secondary outcomes

In order to evaluate the impact of a SPIP or DPIP on the QoR, we will also reassess the QoR-15 score at 48 h (H48) and 72 h (H72) after the surgery. We will compare analgesic efficacy at rest and during exercise (cough, physiotherapy, mobilization) between the different groups via a pain intensity evaluation using a numeric rating scale (NRS, 11 items, 0 for no pain and 10 for the maximum pain) up to H72 after the surgery and during the removal of the drains at 24 h after surgery (H24). We will specify the proportion of painful patients (defined by an NRS value > 3) at H24 and H48. We will also collect the main area of pain during the first 24 h after surgery between the back, the head, the thorax, the abdomen, and the saphenous scar. We will also compare the postoperative consumption of analgesics (both morphine equivalents and non-opioid drugs) up to 5 days after the surgery.

The tolerance of the technique will be compared by assessing the proportion of patients with at least one local complication of regional anesthesia: systemic intoxication to local anesthetics within 3 h after injection, hematoma or infection at the puncture site during the 30 days after the surgery (D30). We will also compare in each group the proportion of patients with at least one major hospital postoperative complication [25] (the complete list is presented in Table S.2 in the supplementary materials).

We will assess in each group the proportion of patients with a return to preoperative weight, as the proportion of patients with bowel movements assessed on days 3 and 5 after surgery. We will compare the delay of extubation (measured between the admission in ICU and the first attempt of extubation), the length of stay in ICU, and the length of hospital stay censored at D30 if the patient is still hospitalized. The readmission rate in ICU will be specified at D30.

The anesthetist’s satisfaction with the ease of performing the technique (i.e., DPIP or SPIP) will be evaluated on a 5-item Likert scale (not at all satisfied, not very satisfied, neutral, fairly satisfied, very satisfied) and compared between the two intervention groups with blocks.

### Sample size

In the database used to validate the FQoR-15 questionnaire, 21 patients were operated on for cardiac surgery with sternotomy, and the average FQoR-15 score was 90 ± 18. The minimal important difference of the QoR-15 score is usually set at 8.0 [26].

We assume that the DPIP has a greater impact than the SPIP on QoR, and we set mean QoR-15 values of 98 in the SPIP group and 106 in the DPIP group.

**Fig. 3:**
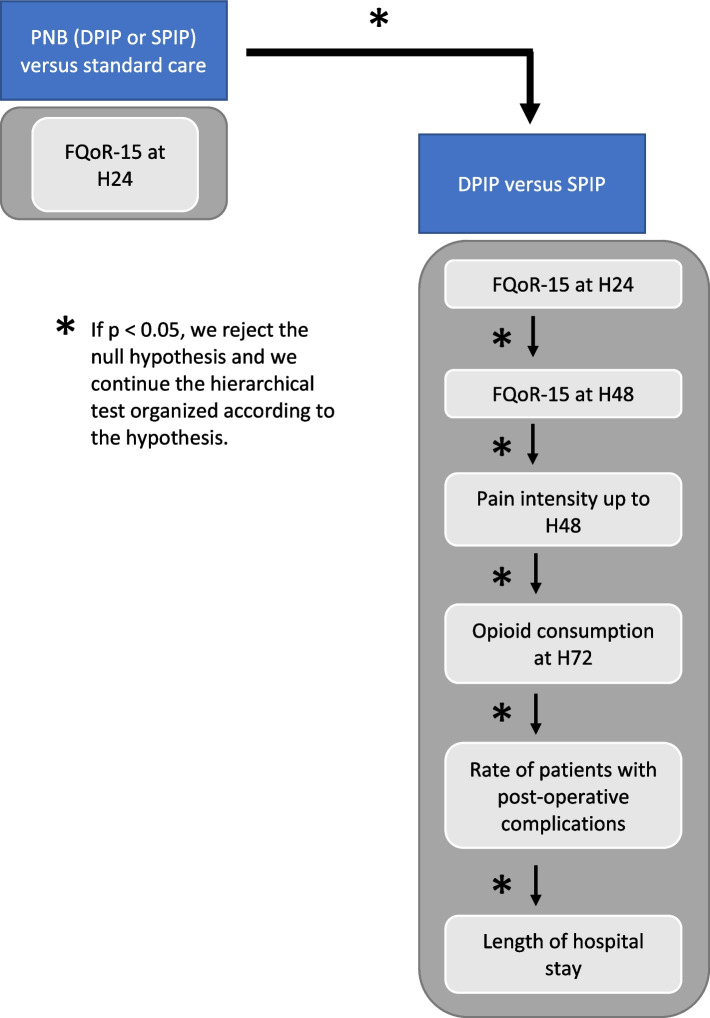
Diagram of the hierarchical classification of the statistical tests (type I error management). Abbreviations: Deep PIP, deep parasternal intercostal plane block; FQoR-15, French version of the QoR-15; H24/48/72, 24/48/72 h at surgery; PNB, peripheral nerve block; Superficial PIP, superficial parasternal intercostal plane block

The management of comparisons and the type I error risk will be performed in a sequential hierarchical strategy (Fig. 3). First, we will compare the primary endpoint (QoR-15 value at H24) between the interventional groups (patients in the SPIP and DPIP groups combined) with the standard group. If efficacy is revealed by this first comparison, further comparisons will be performed between the SPIP and DPIP groups only. Considering a type I error risk of 0.05 in a two-sided test and a statistical power of 0.8, 80 patients will be needed in each intervention group (i.e., SPIP and DPIP) to obtain the minimum power to conclude on the comparison between both groups. With 80 patients in the standard group, we expect to achieve greater than 95% power in the first analysis of the primary outcome (interventional groups versus standard group). Considering our three groups (SPIP, DPIP, and standard groups) and assuming a maximum rate of 5% of patients for whom the primary endpoint will not be available, we plan to include 254 randomized patients.

### Follow-up

Day 0 (D0) is the day of surgery with hour 0 (H0) corresponding to the time of randomization. The delay between randomization, achievement of the intervention for the arms with PNB, and admission to the ICU will be negligible (a few minutes). Ten visits are scheduled after the inclusion, and the summarized plan is presented in Fig. 1. The follow-up will begin at the pre-inclusion visit during the anesthesia consultation aiming to collect demographic data, type of surgery, medical history, vitals parameters (cardiac rate and blood pressure), and the pre-operative QoR-15 score.

The inclusion visit will be realized at the end of the surgery during the skin closure. Once the patient is included and allocated to one arm, the block will be carried out according to the allocation group before the transfer to the ICU. The operator’s satisfaction will be assessed on a Likert scale at this time. Perioperative surgical and anesthesia data will be recorded.

On ICU admission, the collected data will be the hemodynamic parameters, the biological values, and a local or systemic complication possibly linked to block. The intensity of pain at rest and during effort, the consumption of analgesics, and the notification of local or systemic complications will be collected during the following visits at 3, 6, 12, 24, 48, and 72 h after the surgery. The QoR-15 questionnaire will be completed at H48 and H72. At H24, patients will detail the body localization of the most intense pain, the use of a rescue peripheral nerve block, and the intensity of pain at the removal of the pericardial drains. Morphine equivalents and non-opioid analgesic drugs consumptions will be collected at 4 and 5 days after the surgery. The patient’s weight and the occurrence of bowel movement will be noted at H72 and 5 days after the surgery.

Thirty days after the surgery, the following data will be collected: a puncture site or a postoperative complication, the length of hospital censored at D30 if the patient is still hospitalized, and length of stay in ICU. Potential readmission to ICU will also be notified. If the patient’s condition does not allow him to be contacted directly or by phone, the person in charge of the follow-up will be contacted (e.g., family member or physician).

### Safety

The major potential adverse effects linked to SPIP and DPIP are pneumothorax, mediastinitis, hematoma, puncture site infection, and intoxication with ropivacaine [27–29]. Continuous monitoring of vital parameters within 3 h following the PNB procedure will be carried out to detect local anesthetic toxicity. A chest X-ray is systematically realized at ICU admission, and this investigation will allow to detect the presence of a pneumothorax. The body temperature will be noted every 3 h by a nurse, and the monitoring of the occurrence of complications at the puncture site will be done at each visit up to D30. The occurrence of a serious adverse event will be collected at any time during the patient’s follow-up (up to 30 days), whose imputability with one of the allocation groups will be discussed.

### Data collection and study monitoring

The information will be recorded in the electronic web-based case report form (Ennov Clinical® eCRF) managed by the clinical research team of the Angers University Hospital.

A clinical research associate (CRA) mandated by the study sponsor will ensure the successful completion of the study, the data collection, documentation, recording, and report, in accordance with the Standard Operating Procedures implemented in the Angers University Hospital and in accordance with good clinical practice, laws, and regulations.

Different items will be reviewed for every fifteen included patients: signed informed consent, compliance with procedures, and quality of collected data. An automated data check will be made by the data management team based on the data validation plan signed by the coordinating investigator. Detected errors will lead to the issuance of requests for information and electronic correction. Given the low risks associated with this study, which consists of comparing two treatments already used routinely, an independent monitoring committee will not be set up for this study.

The promoter can notify any deviation from the protocol using a breach report form.

### Type I error management

The management of the type I error will be done in a sequential hierarchical manner. A representative diagram of the hierarchical decision is presented in Fig. 3. As long as the *p*-value is less than 0.05, the comparisons will be continued for conclusion. If for any of the comparisons the *p*-value is superior to 0.05, the comparisons will only be made for exploratory purposes and will be delivered explicitly as such. The hierarchical list of comparisons will be made first on the primary outcome (QoR-15 at H24) between the interventional groups and the standard group. The rest of the comparisons will be done between the SPIP versus DPIP groups on the primary endpoint and then on several secondary endpoints. Comparisons on other outcomes (not shown in the diagram) will be exploratory.

### Statistical analysis

All analyses will be performed using the R software (version 4.1.3). The main analysis will be in intention-to-treat, including all randomized patients. Patients will be analyzed according to their randomization group. A per-protocol analysis will be performed as a sensitivity analysis to assess the main robustness analysis. A flow chart of all patients (Fig. 2) and descriptive statistics will be used to describe baseline characteristics. Data will be presented by their mean with standard deviation, median with interquartile range, according to their normal or non-normal distribution respectively, and number with the percentage of sample (%). For the endpoint comparisons, we will report both absolute measures (differences in means or percentages) and relative measures (odds ratios or hazard ratios), with their 95% confidence interval.

We will detail the missing data on the endpoints involved in the type I error hierarchical management only. We will identify the status of these missing data for these criteria between completely randomly missing, randomly missing, or non-randomly missing. For criteria with less than 10% missing data, we will not perform a missing data management method. For criteria with more than 10% missing data on these criteria, we will perform sequential multiple imputations by chained equations (mice package in R, with 5 imputations, from predictors/variables at inclusion, set.seed 1111). If the multiple imputation method is used (i.e., more than 10% missing data), we will study the impact of the data imputation. We will then carry out an analysis of the concrete cases, as well as the following sensitivity analyses: “best–worst” and “worst-best.” For the “best–worst” scenario, it is assumed that all patients lost to follow-up will have a beneficial outcome, whereas in the “worst-best” scenario, they will have a negative outcome. For the continuous outcomes, the beneficial outcome will be the mean value of the group plus one standard deviation, while the adverse outcome will be the mean minus one standard deviation.

For all analyses using regression models, the included covariates will be the allocation group, as well as the stratification group (surgery with or without thoracic internal arterial graft). For analyses requiring a mixed model, the covariates introduced will be a fixed effect for the treatment arm, a fixed effect for the stratification factor, a fixed effect for the time frame of the visit, and a random effect for the patients, and under an unstructured variance/covariance matrix. If the mixed models do not converge, we will use a compound symmetry correlation structure. To address the main objective, we will use a linear regression model. Similarly, the QoR-15 score at H48 will be analyzed using a linear regression model. The evolution of effort pain up to H48 will be analyzed using a linear mixed model. The evolution of morphine equivalent consumption will be analyzed using a mixed model, following a Poisson distribution. The rate of occurrence of at least one postoperative complication will be compared by logistic regression. Censored 30-day hospital stay will be assessed by survival analysis using a semi-parametric Cox model.

The other endpoints will not have a method of imputation for missing data. Thus, only those individuals for whom endpoints are available will be included in the analyses. These data will be analyzed for exploratory purposes. The evolution of pain at rest will be analyzed with a linear mixed model, and we will compare the evolution of the consumption of non-opioid analgesic drugs with a mixed model, following a Poisson distribution. The proportions of patients with pain at H24 and H48, as the rate of local and systemic complications during the follow-up will be compared using logistic regression. The total hospital consumption of morphine equivalent in the first 72 h will be assessed by Poisson regression. Pain intensity at the pericardiac drain removal at H24 will be assessed by linear regression. Time to resuscitation censored at 30 days, time to extubation, time to recover bowel movements, and time to return to preoperative weight will be assessed by survival analysis using a semi-parametric Cox model. The distribution of postoperative complications will be compared by multinomial regression. Satisfaction with the block procedure and the main painful area at H24 will be presented descriptively in the groups.

All tests will be two-sided. Statistical significance is set in the usual way with a *p*-value < 0.05, and 95% confidence intervals will be estimated for each calculated value. For the primary endpoint (QoR-15 score at H24), a sensitivity analysis will be performed without including the stratification factor in the model.

## Discussion

The potential benefits of using chest wall blocks in cardiac surgery was highlighted in recent trials. Concerning the SPIP, Zhang et al. found decreases in length of hospitalization, postoperative pain at mobilization and at rest, and even in postoperative analgesic consumption when comparing SPIP to a placebo [30]. These results were corroborated on the pain measured after extubation [31]. Regarding the DPIP, a study highlighted the impact of the technique to reduce postoperative pain, length of hospitalization, and consumption of analgesics within 48 h [15]. This trend was also found in cardiac surgery in the pediatric population [32, 33].

Our study should provide interesting additional data to confirm whether these chest anterior wall blocks are useful for decreasing postoperative pain and improving recovery and then to compare SPIP and DPIP for these criteria. To evaluate the interest of these blocks, we choose the primary criteria focused on patient’s early recovery. Recent international consensus (i.e., the SteP-COMPAC group) highlighted the importance of this patient-centered endpoint in perioperative medicine [23]. The QoR-15 has already shown its reproducibility, sensibility, and ease to use to reveal the quality of postoperative recovery in a unidimensional approach. QoR-15 is increasingly used as an endpoint in randomized trials in anesthesia [24].

If the hypotheses on the effectiveness of the SPIP and/or DPIP are confirmed for the early QoR, associated benefits could be a better patient experience, a lower incidence of postoperative complications (particularly related to opioids consumption), and even an impact on the lengths of hospital stay.

To summarize, this study will compare two types of chest anterior wall blocks versus a standard care with no regional anesthesia in patients scheduled for cardiac surgery with sternotomy, on their early QoR, but also on their pain status. The findings will also provide data on potential local and general complications that could be attributed to these invasive techniques, helping us to evaluate the benefit/risk ratio of the use of chest wall blocks for cardiac surgery.

## Trial status

This study was approved by the French Institutional Review Board “North-West IV” (Lille, France, number: 2022-A00238-35) and the National Agency for Drug and Health Product Safety and registered at ClinicalTrials.gov (NCT05345639) on April 26, 2022. The recruitment of participants started in July 1, 2022. The anticipated period is 18 months.

## Post-trial care and dissemination plan

There is no anticipated harm and compensation for trial participation. The dissemination plan will consist of the publication of the results in a A-rank journal once the study is completed as well as the presentation of the study at national, European, and international congresses.

## Supplementary Information

**Additional file 1: Table S.1.** Pain management protocol in the intensive care unit. **Table S.2.** List of collected complications during the 30 days after surgery.

## Data Availability

The data generated could be shared after a request to the corresponding author. Three years after the end of follow-up of the last included patient, we will deliver a completely deidentified data set to an appropriate data archive for sharing purposes.

## Abbreviations

CRA: Clinical research associate
D0/30: 0/30 Day(s) at surgery
Deep PIP: Deep parasternal intercostal plane block
FQoR-15: French version of the QoR-15
H24/48/72: 24/48/72 Hours after surgery
IV: Intravenous
ICU: Intensive care unit
MAC: Minimum alveolar concentration
NRS: Numerical rating scale
PCA: Patient-controlled
PNB: Peripheral nerve block
QoR: Quality of recovery
QoR-15: Quality of Recovery-15 score
SFAR: French Society of Anesthesia and Intensive care Medicine
Superficial PIP: Superficial parasternal intercostal plane block
TIVA: Total intravenous anesthesia
