# Supplementary material for: Effect of superficial and deep parasternal blocks on recovery after cardiac surgery: study protocol for a randomized controlled trial
Source: Trials. 2023 Jul 6;24:444. doi: 10.1186/s13063-023-07446-2 (PMC10324106; doi:10.1186/s13063-023-07446-2)
Supplement: Supplementary file 1 — Additional file 1: Table S.1. Pain management protocol in the intensive care unit. Table S.2. List of collected complications during the 30 days after surgery. [file 13063_2023_7446_MOESM1_ESM.docx]

**Table S.1.** Pain management protocol in the intensive care unit.

**PARACETAMOL**

- 1g x 4 per day systematically (intravenous initially, then orally as soon as possible)

**MORPHINE titration**

- 2 mg every 5 min to obtain a pain score < 4 on a numerical rating scale (NRS) of pain from 0 to 10
- Before the start of the intravenous patient-controlled analgesia

**PCA (patient-pontrolled analgesia)**

- Using oxycodone or morphine
- Bolus of 1 mg, refractory period of 7 minutes, maximum dose of 20 mg over 4 hours.
- Usually maintained until 24-48 hours after the surgery then switched by short-acting sublingual oxycodone, 5 mg every 4 hours if NRS > 3, and/or by oral long-acting oxycodone, 10 mg x 2 per day if daily infusion on PCA > 20mg

Molecules of recourse in case of ineffective analgesia

**KETOPROFEN**

- 100 mg every 12 hours intravenous or 50 mg every 8 hours orally
- Contraindications: > 75 years, GFR < 60 mL/min/1,73m^2^, diuresis in the first 3 postoperative hours < 0.5 ml/kg/h, abnormal bleeding per-/post-intervention, per-intervention lactates > 3 mmol/l

**NEFOPAM**

- 20 mg every 6 hours intravenous
- Contraindications: angle-closure glaucoma, benign prostatic hyperplasia, confusion, comitiality, > 75 years

**Table S.2.** List of collected complications during the 30 days after surgery.

**Major postoperative complications :**

- De novo postoperative arrhythmia : atrial fibrillation, flutter, ventricular tachycardia, ventricular fibrillation
- De novo postoperative conduction disorder: atrioventricular block with or without the need for electro-training, branch block
- De novo postoperative repolarization disorder: ST elevation, ST depression, T wave inversion
- Revision surgery (for bleeding, tamponade or pericardial effusion);
- Respiratory failure (defined by the need for prolonged mechanical ventilation > 24 hours or the need for orotracheal intubation);
- Mediastinitis (defined by surgical revision for lavage with positive intraoperative bacteriological sample);
- Neurological complication (defined by the occurrence of an ischemic or hemorrhagic cerebrovascular accident documented on CT or MRI);
- Acute renal failure requiring hemodialysis;
- Gastrointestinal complication (defined by upper or lower digestive bleeding, postoperative ileus without resumption of stool transit after 5 days or mesenteric ischemia);
- Infectious complication with bacteraemia;
- Pneumothorax (confirmed by chest X-ray or CT scans);
- Infectious pneumonitis (defined by an increase in oxygen demand, clinical signs of sepsis with pulmonary bacteriological confirmation)

**Chest wall blocks-related complications**

- Local anesthetic toxicity defined by the occurrence of at least one of the following symptoms within 3 hours of performing the PNB: perioral paresthesia, numbness of the tongue, tinnitus, hyperacusis, dizziness, visual disturbances, dysarthria, hypoaesthesia, tremor, muscle contraction
- Block site hematoma
- Infection at the puncture site
- Mediastinitis
- Pneumothorax
